# Routine patient surveys: Patients’ preferences and information gained by healthcare providers

**DOI:** 10.1371/journal.pone.0220495

**Published:** 2019-08-01

**Authors:** Andrea C. Schöpf, Werner Vach, Marcel Jakob, Franziska Saxer

**Affiliations:** 1 Section of Healthcare Research and Rehabilitation Research, Faculty of Medicine and Medical Center, University of Freiburg, Freiburg, Germany; 2 Department of Orthopaedics and Traumatology, University Hospital Basel, Basel, Switzerland; Medical University Graz, AUSTRIA

## Abstract

**Background:**

Patient feedback after contact with a hospital is regarded as an important source of information for the improvement of local healthcare services. Routine patient surveys are in widespread use to obtain such feedback. While general principles for the composition of this kind of surveys have been described in the literature, it is unknown which method of contact and topics of feedback are important to patients in postcontact healthcare surveys.

**Material and methods:**

We invited 2931 consecutive patients who had in- or outpatient contact with the Department of Orthopaedics and Traumatology at the University Hospital Basel to an anonymous survey. They were asked whether they were generally in favor of feedback surveys. They also had the opportunity to state their preferred form of contact (text message, app, email, online or letter) and provide up to three topics that they regarded as specifically important in patient surveys.

**Results:**

A total of 745 patients participated in the survey (25.4%), of these 61.9% expressed the preference to be surveyed, and 69.1% selected `letter’ as one of the preferred forms of contact. Favoring only `letter’ contact increased substantially with age. Overall 54.6% of patients stated at least one topic that they wished to give feedback on. The most frequent topics were related to treatment and rather general aspects regarding staff and overall impression. The wish to include suggestions for improvements was rarely mentioned as specific topic.

**Conclusions:**

The majority of patients seem to be rather indifferent to the existence and content of patient surveys. They mention a wide range of topics from general to specific ones, but do not express interest in the opportunity to suggest changes. There is a need to effectively engage patients in healthcare planning using new approaches to obtain valuable feedback on patients’ hospital stay and contact experiences. These new approaches should ideally be more informative and cost-effective than the current practice.

## Introduction

Hospital discharge surveys or surveys after contact with out-patient healthcare services (post-contact surveys) are an important instrument for quality control and benchmarking. The ultimate goal of this routine patient feedback is the improvement of services. To achieve this goal several conceptual and organizational aspects regarding the development and implementation of surveys need to be considered.

One aspect is what kind of feedback is actually measured. In the current literature several expressions are used sometimes interchangeably for similar multidimensional concepts [1]: ‘patient experience’, ‘patient perception’ and ‘patient satisfaction’.

For the purpose of this article the three terms will be distinguished as follows

- Patient experience refers to what actually happened during the hospital stay and how the patient reports it implying a rather objective account on healthcare provision, e.g. “Did the surgeon inform you about potential risks?”
- Patient experience is sometimes also extended to address the degree to which patients think their needs are met [2].This aspect of whether needs are met will be referred to as patient perception [3], e.g. “Did you receive enough information to make a decision?”
- Patient satisfaction is commonly seen as a result of patients’ expectations, preferences and experiences [4]. It focuses on the subjective evaluation of the hospital stay, i.e. how the patient feels [5]. E.g. “Were you happy with the information you received from your surgeon?”

These definitions do not merely reflect a distinction without difference; Jenkinson et al. [6] reported conflicting results as half of the respondents gave excellent scores for their hospital stay despite reporting problematic experiences in 10% of the areas covered by the survey. Similarly Bjertnaes et al. [7] concluded that patient-reported experiences and overall satisfaction are related but yet distinct from each other. Fenton et al. [8] even found a positive association between patient satisfaction and increased mortality as typically employed quality indicator, while Glickmann et al. [9] concluded that patient satisfaction was associated with quality of care. Accepting that the various concepts capture various aspects contributing to the multifaceted construct of healthcare quality, these results underline the importance of designing surveys according to a specific purpose as well as the importance of analyzing potential confounders.

A second aspect is the selection of content areas, as determined by the purpose of the survey that can validly be evaluated by patients or other target populations. The NHS (National Health Service) has defined a relatively comprehensive list of domains of “good” patient experiences (NHS Patient Experience Framework [10]). Patients can perceive and judge these domains with relative ease, which can advance the development of hospital discharge surveys.

- Respect for patient-centered values, preferences, and expressed needs
- Coordination and integration of care across the health and social care system;
- Information, communication, and education
- Physical comfort
- Emotional support and alleviation of fear and anxiety
- Welcoming the involvement of family and friends
- Transition and continuity
- Access to care with attention to organizational aspects such as waiting times

As a third aspect, organizational issues are relevant. To obtain reliable results, a high return rate is one important aspect. An overview of 210 studies on patient satisfaction demonstrated a mean response rate of 72.1% (25–98%) [11]. The problem of low response rates continues in more recent research [12,13,14]. The issue of potentially low response rates is connected to the problem of systematic nonresponse. Both can create bias and, in turn, uncertainty regarding the generalizability of the results [15,16]. There is evidence that non-responders or late responders tend to be less satisfied with their healthcare provision or evaluate their experiences more negatively [17,18] which in turn would overestimate satisfaction, particularly for providers or institutions with low patient satisfaction [19].

Therefore, a high response rate is crucial to draw valid conclusions. [10] reported response rates for patient satisfaction interviews to be higher than for self-report questionnaires, but this was not confirmed by [9]. The latter authors though found, that the response rate was higher if recruitment and/or data collection were performed face-to-face rather than by mail. Considering survey practice, a patient tailored approach for the distribution of questionnaires (spectrum from face-to-face distribution, mailed letters to use of apps on mobile devices) as well as a regulated distribution practice (i.e. quantity of surveys per contact and per contacted service) seem to support the generation of meaningful data.

The consideration of all these aspects in routine surveys and the implementation of sustainable systems for corrective actions might pose a challenge to healthcare providers and organizations. In the process of reorganizing the routine patient survey practice in our institution, we conducted a specific survey in a consecutive patient population after contact with our hospital to evaluate patients’ organizational and conceptual preferences considering postcontact surveys.

## Materials and methods

After evaluation by the competent ethics committee (Ethikkommission Nordwest- und Zentralschweiz, Ref. 2017–01845), documenting no need for a formal ethical approval according to Article 2 of the Swiss Human Research Act (HRA), but demanding the adherence to general ethical research principles according to HRA Article 51, paragraph 2, the study was carried out following applicable law, the principles of good clinical practice (GCP) and the Declaration of Helsinki.

All patients (out- and in-patients) who had contact with the Department of Orthopaedics and Traumatology of the University Hospital Basel between August and September 2016 were invited to an anonymous survey assessing their preferences for postcontact surveys. All patients were contacted by postal mail on April 26^th^ 2017 with a cover letter explaining the project and a paper survey form. Additionally, a link and a QR (Quick Response) code were offered, allowing the completion of the survey via the online SurveyMonkey portal. The survey was conducted in German, and patients who specifically asked for a translation were provided with a French version. Other languages were not demanded. The survey stayed open for approximately 10 weeks until July 15^th^, 2017. A response by a patient to the invitation was regarded as an implicit consent.

The original survey form is displayed in S1 Fig. Patients were initially asked to provide basic demographic data (age and gender). Question 1 addressed the principle question of whether patients wish to be surveyed by the hospital after their stay and if so whether the survey should focus on the treatment result, satisfaction with the hospital or both aspects. Question 2 asked about the preferred form of contact for a potential survey, allowing the choice of one or several of the options: “text message”, “app”, “email”, “online (internet)” or “letter”. Question 3 was an open-ended question that gave the patients the possibility to state three topics that they regard as important to be covered in a patient survey. Question 4 asked the number of surveys, patients had received within the last 6 months from the university hospital, including the survey presented in this article.

For the analysis of the statements in response to Question 3, one of the researchers (AS) created a set of categories, consisting of 11 main categories and 26 subcategories (S1 Table). If a patient specified several topics per row (instead of only one per row, as intended), they were counted and classified as separate statements. The categories and the assignment of the individual statements to the respective categories were reviewed by two of the other authors (WV, FS) and by two additional researchers. Disagreements were discussed and subsequently resolved.

The results are reported as absolute numbers and percentages, and they are visualized using bar charts. Frequencies of categories are reported at the patient level, i.e., they refer to the number of patients mentioning at least one topic of the specific category. A latent class analysis was applied to the subcategory data in order to identify subgroups of patients with specific profiles regarding thematic priorities. The Akaike Information Criterion [20] was used to determine the number of classes.

## Results

### Response rates and patient characteristics

**Fig 1:**
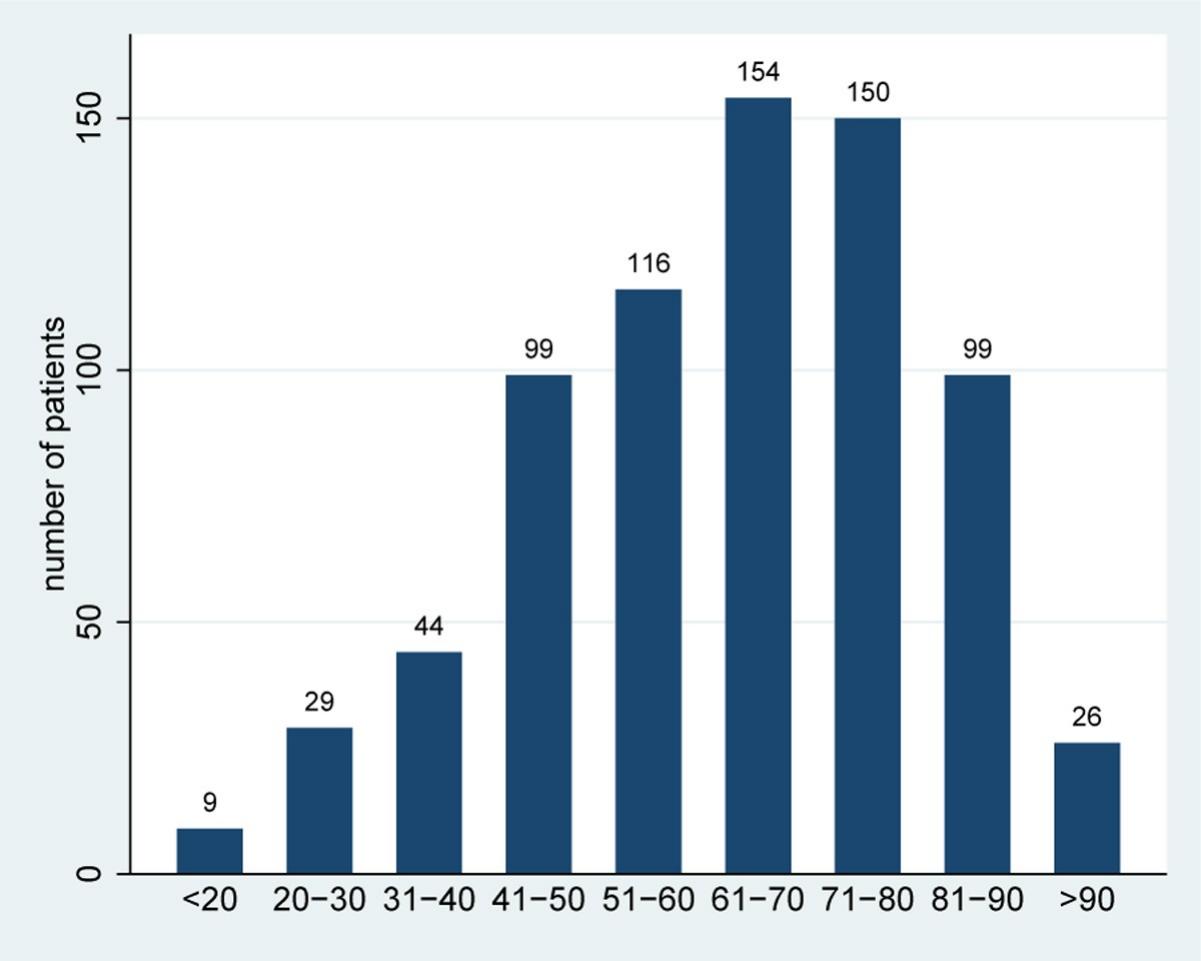
Age distribution of the participating patients who stated their age.

In the 2 months of observation 2931 patients were seen in our department and were contacted by mail. Overall, 87 patients did not receive the invitation: 78 were not traceable, 8 were reported deceased, and 1 refused the letter. There were no incoming responses after the official closure date of the survey. We received a total of 745 responses (25.4%), with 52 participants using the online form. A total of 654 responders reported their gender, of which 367 (57.5%) were female. Age was stated by 735 participants, resulting in the age distribution shown in Fig 1. The joint distribution of age and gender is shown in S2 Table including the proportion of respondents compared to the target population. A total of 575 patients reported the number of surveys received within the last 6 months, which ranged from 0 to 7 with an average of 1.1 and a median of 1 (without the current survey).

### Wish to be surveyed and preferred focus of content

**Fig 2:**
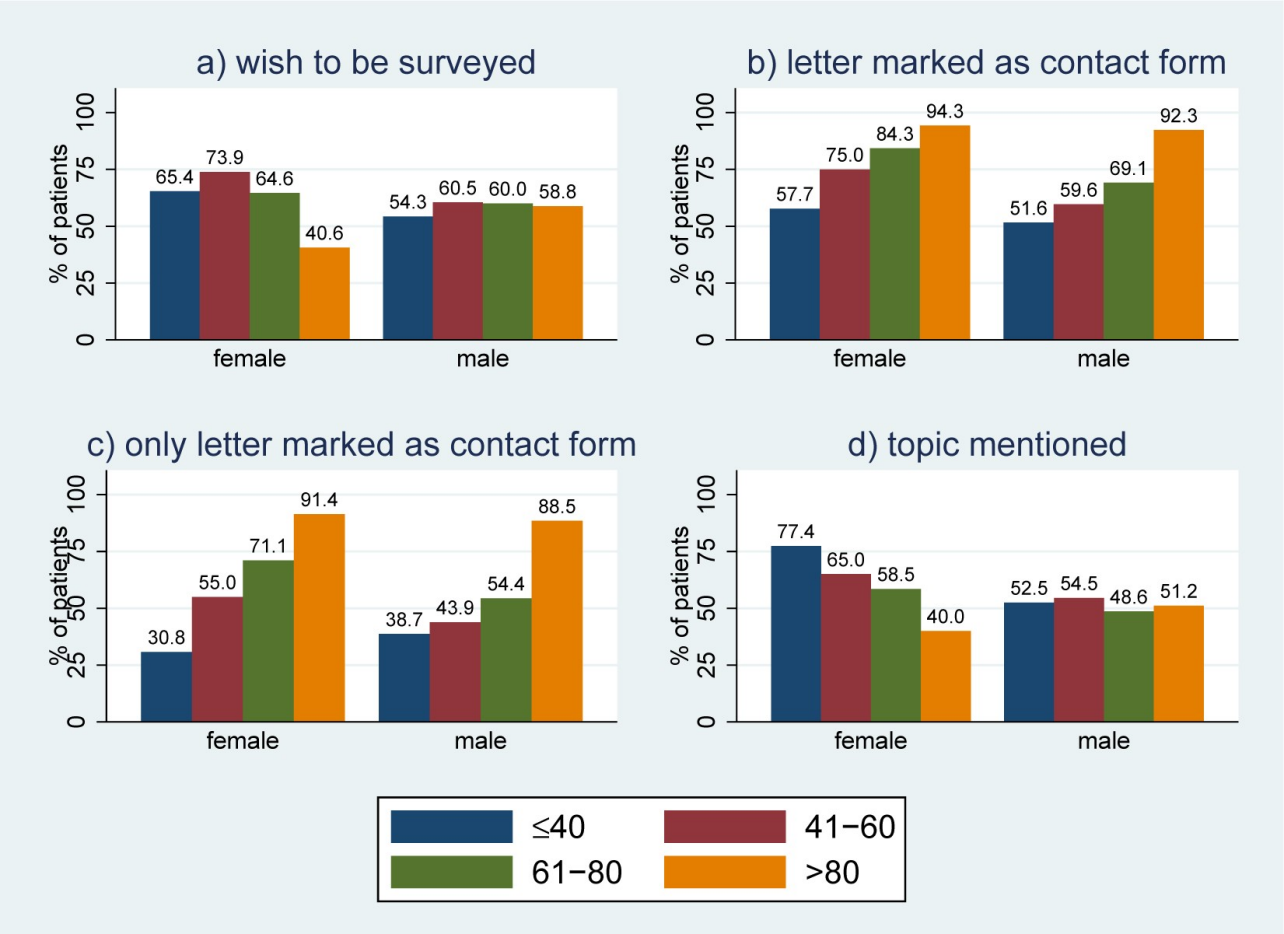
The distribution of a) “wish to be surveyed”, b) “letter” marked as contact form, c) only “letter” marked as contact form, and d) “topic mentioned” according to age and gender.

Question 1 was answered by 640 patients, of whom 396 (61.9%) wished to be surveyed by the hospital in principle. The rates were similar for females (205/327, 62.7%) and males (145/245, 59.2%) (Fig 2A). Overall, 391 participants also indicated their preferred thematic focus: 251 (89.8%) chose “treatment and satisfaction”, 34(7.7%) only “satisfaction”, and 10 (2.6%) only “treatment”.

### Form of contact

Question 2 was answered by 499 patients. A majority of 345 patients (69.2%) preferred “letter” as form of contact, followed by “email” (n = 143, 28.7%), “online” (n = 120, 24.1%), “text message” (n = 16, 3.2%) and “app” (n = 14, 2.8%). The choice of “letter” as one possible form of contact increased with age (Fig 2B). The relation between the choice of letter and age was more pronounced if “letter” was chosen as only form of contact (Fig 2C). Overall, 276 patients (55.3%) marked “letter” as the only form of contact, and the fraction increased with age for both females and males; in patients between 40 and 80 years of age, higher rates can be observed in females compared to male responders.

### Choice of topics

A total of 407 patients mentioned at least one topic (54.6%) as preference for potential surveys. This rate decreased in females from 77.4% in those younger than 40 to 40.0% in those older than 80, but was rather constant at approximately 50% in males (Fig 2D). The majority, i.e. a total of 291 (71.5%) of the 407 patients gave three answers, 69 (16.9%) gave two answers, and 47 (11.6%) gave one answer. On average, responders mentioned topics from 2.4 different main categories and from 2.8 different sub categories (S1 Table).

**Fig 3:**
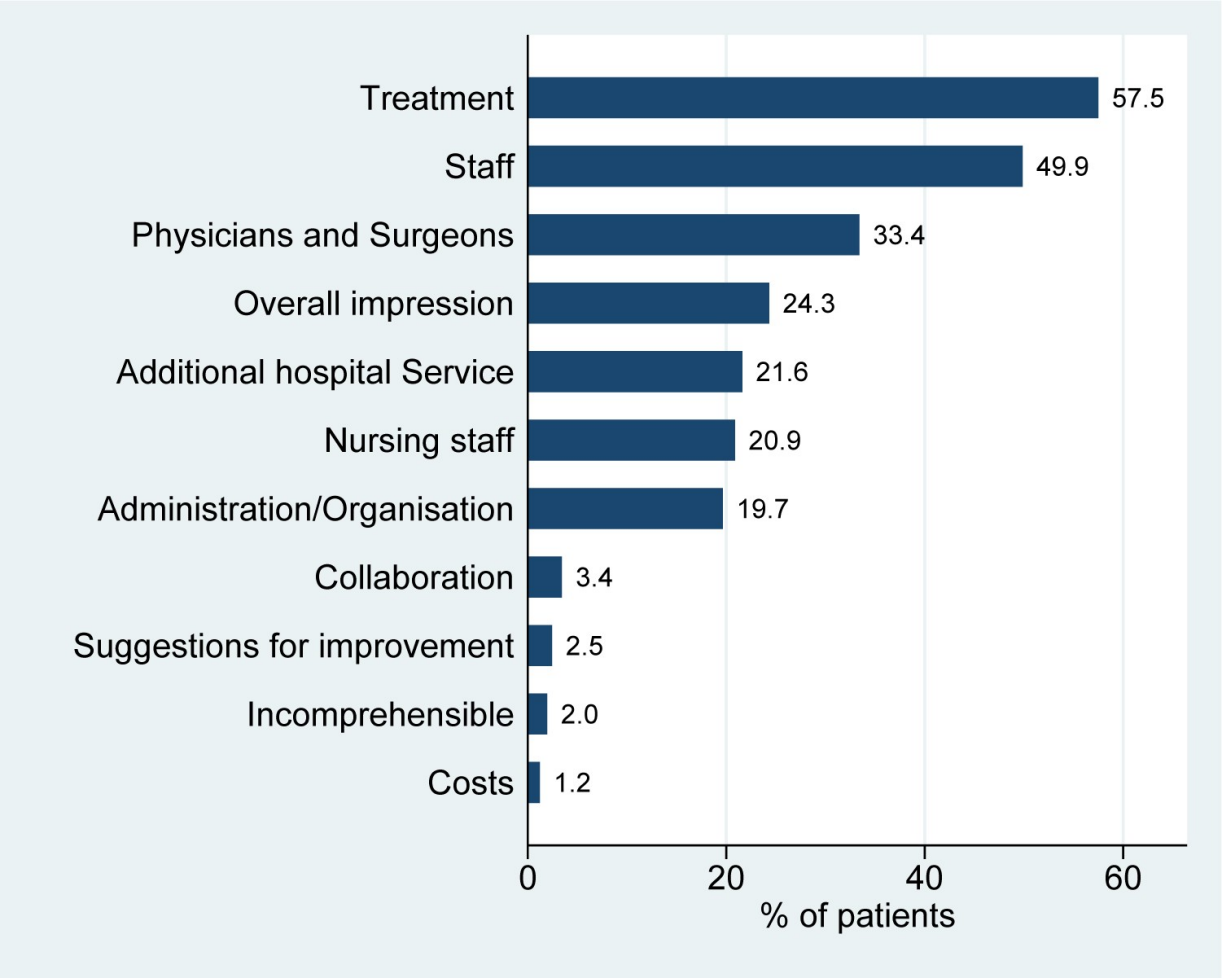
Distribution of choice of topics–main categories. The figure shows the relative frequency of participants mentioning a given main category among the topics reported within all participants.

**Fig 4:**
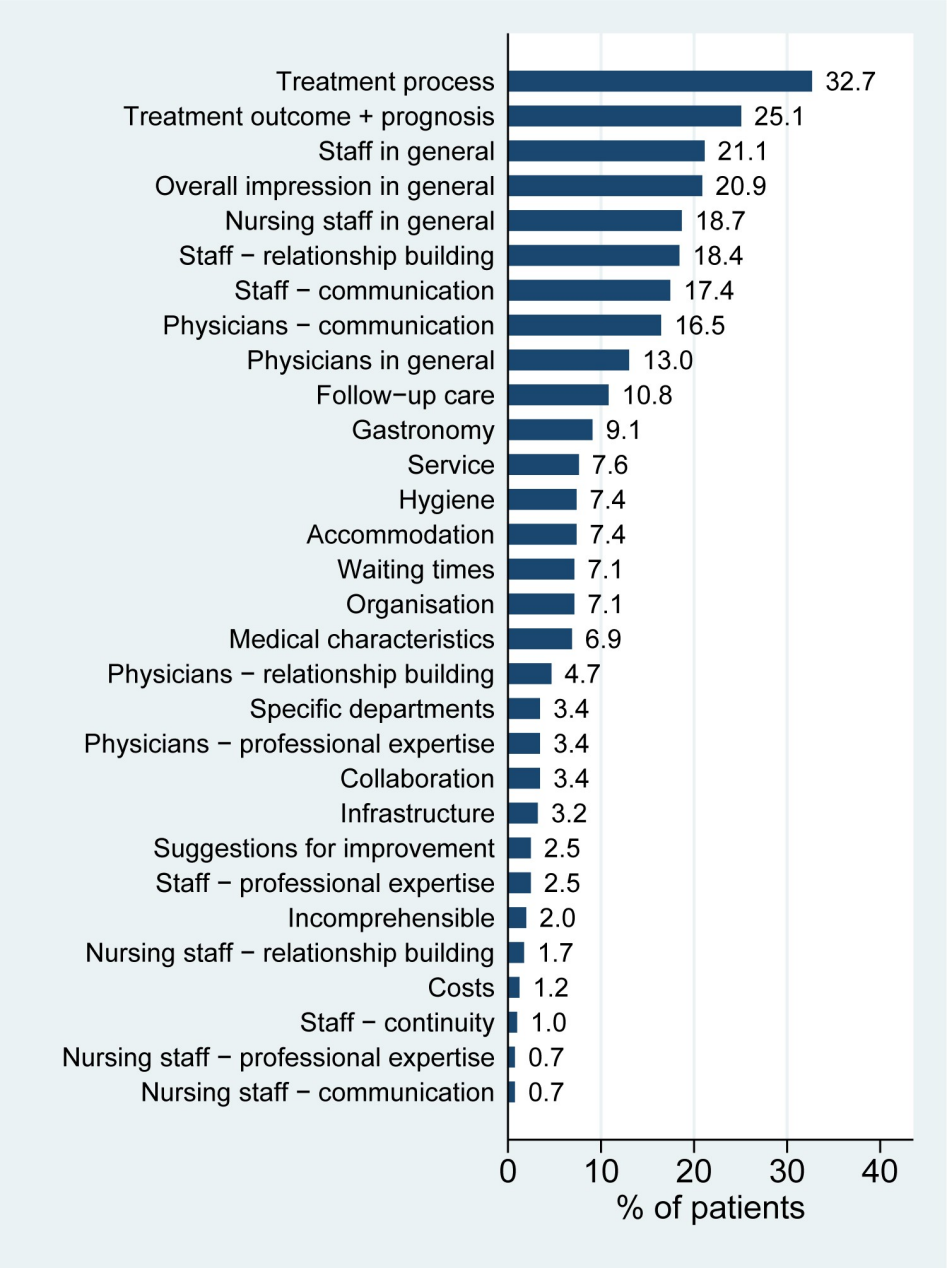
Distribution of choice of topics–sub categories. The figure shows the relative frequency of participants mentioning a given sub category among the topics reported within all participants.

Figs 3 and 4 show the frequencies of the main categories (as defined in S1 Table) at the patient level. We can observe, that the most frequently mentioned main category is `Treatment’. The high frequencies of the main categories `Staff`, `Physicians and surgeons’ and `Nursing staff’ together, however, underline the importance of the human aspect in hospital stays. Very few patients stated “suggestions for improvement” as a topic.

Figures in the Supporting Information present the same numbers stratified by gender and age. At the level of the main categories, we observe no relevant differences between males and females (S2 Fig), but in younger patients there is a tendency towards a stronger focus on staff and administrative issues (S3 Fig).

At the level of the subcategories, we observe that males emphasize `Treatment outcome and prognosis’, `Overall impression’ and `Gastronomy’; females have a stronger focus on `Staff in general’ (S4 Fig) than males. Again there are differences in priorities between age groups. While younger patients underline the importance of `Staff in general’, `Waiting times’, `Physician–relationship building’, older patients have a stronger focus than younger patients on `Physicians in general’, `Physicians–communication’, and `Medical characteristics’ (S5 Fig).

### Patient profiles based on topics

**Fig 5:**
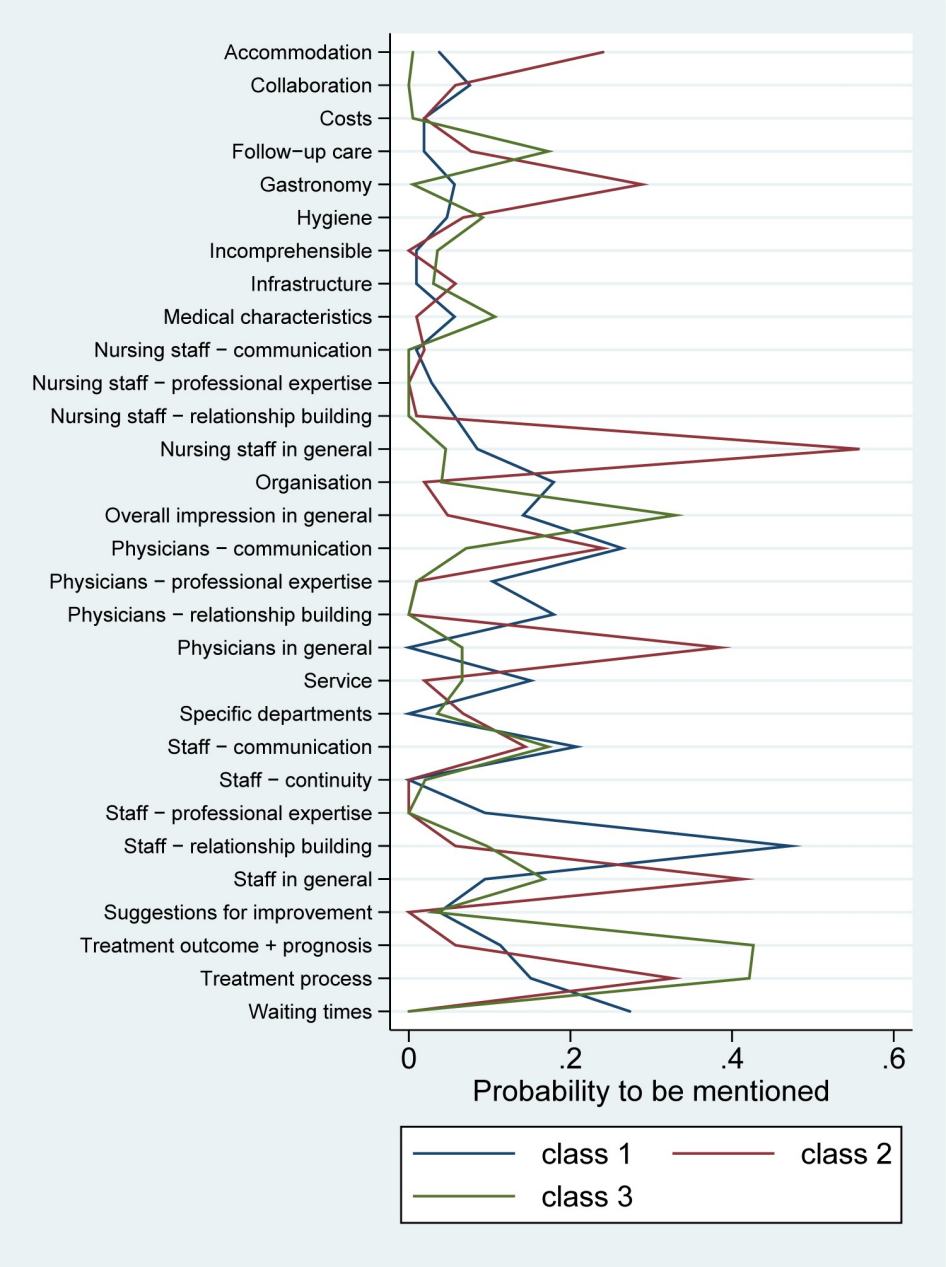
Profile plot of results from the latent class analysis. The figure shows the estimated probability of mentioning a given subcategory for members of the three different classes identified by the latent class analysis.

The latent class analysis suggests distinguishing three groups of patients with different profiles with respect to mentioning certain topics (Fig 5).

The first group comprising 31% of the responders, is characterized–in comparison to the other groups–by a superior interest in the topics `Physician–professional expertise’, `Physicians–relationship building’, `Staff–relationship building’, `Organization’, `Waiting times’, and `Service’.

The second group comprising 26% of patients, is characterized by increased interest in the topics `Physicians in general’, `Nursing staff in general’, `Staff in general’, `Accommodation’, and `Gastronomy’.

The third group comprising 42% of patients, is characterized by a frequent priorisation of `Follow-up care’, `Medical characteristics’, `Overall impression’, `Treatment outcome and prognosis’, and less frequently `Physician–communication’ and `Collaboration’.

The age distribution was similar in all three groups; however the third group had a higher fraction of males (43.6%) compared to the other two (34.4% and 36.8% in the first and second groups, respectively). We may interpret the first group as patients with a focus on a well-functioning environment (procedural and emotional), the second as patients with a priority on general well-being (staff and hospital service), and the third as patients focusing on the provision of treatment (efficient organization and professional attitude). However, the difference between these groups remains vague for many topics.

## Discussion

### Costs and benefit of routine surveys

Many healthcare institutions use routine patient surveys in order to improve healthcare services. Our results however suggest a limited interest of patients in surveys. In our sample only a quarter of patients responded to the survey, although this would have allowed them to actively participate in future survey design. Of these patients, another 30% state a reluctance to participate in postcontact surveys, which leaves only 18% who want to take part in this form of feedback. This lack of interest in postcontact surveys is much higher than in the aforementioned review on patient satisfaction studies [11].The return rate is however comparable to the hospital’s data from 2017 with a return rate of 36,6% for in-patients (10336/28221) and 28,2% for out-patients (25095/88695) (personal communications with the concerned organizational unit). This limited interest questions the potential benefit of such surveys, particularly with respect to obtaining representative, reliable and useful information.

At our center, the total costs per patient amount to 2.14 CHF (Swiss Francs) per outgoing letter (postage, printing and material) and additional 0.94 CHF per returned letter. Assuming the 2017 quantities for outpatient contact of 1,044,000 patients and inpatient contact of 37,000 patients for the entire organization (annual report for 2017), costs of 2,313,340 CHF would accrue for material and postage alone if patients were to be contacted following every contact, as often happens in routine survey systems. A return rate of 18%–as to be predicted from our survey–adds another 182,905 CHF. A 40% student position for the evaluation of free text feedback adds approximately 24,000 CHF per year, which would amount to a total cost of 2,530,245 CHF (equivalent to 2,537,896 US Dollar or 2,246,441 Euro).

In the current situation, with limited healthcare resources, the cost-benefit ratio of this kind of routine patient survey is insufficient, which implies the necessity of I) finding alternative ways to acquire patient feedback and II) defining clear target areas for the implementation of changes that are included in the survey.

### Response rate and survey technique

One approach to increase the response rate and, thereby, the potential informative value of surveys, might be to offer a choice of various technical means for participation in a given survey. Our results show that more than 50% of patients still prefer the paper format to all digital solutions, especially among those older than 60 years. This corresponds to findings that suggest a limited access to digital infrastructure, lack of technical and digital competence and a reserve towards the “digital world” with old age [21]. Nevertheless, internet use among the elderly is increasing [22], which might change this preference in the future. However, even in the group under 40 years old, 30–40% exclusively want to be contacted by letter. The finding that online survey solutions are not yet fully embraced agrees with several reviews demonstrating a lower response rate for web-based surveys than for other survey methods [23,24]. Reasons for this lower response rates in web-based surveys may include problems with the accessibility, reachability of participants or concerns about confidentiality [25,26]. Nonetheless, an approach combining online and mailed survey techniques seems to be an effective strategy to collect data from young adults [27].

Alternative approaches might be face-to-face distribution of surveys or collection of data as part of the regular discharge process [11]. Disadvantages, however, might be a necessary increase in personnel requirements and the fact, that patients might feel obliged to meet someone’s expectations for reasons such as politeness, gratefulness, perceived authority. The results regarding response rates and face-to-face distribution are not consistent [28,29], but a recent review shows a higher response rate for surveys delivered in-person [28]

Additionally, tablet solutions could be integrated, especially since response rates have been reported to be higher if surveys are completed directly at the facility [30]. The majority of patients (over 80%) reported no problems using tablet computers to complete a survey [31]. It must be taken into account, though, that the level of satisfaction can be influenced by the time of an assessment (i.e. early after contact or late) [30,32]. There are also indications that reminders are an option to increase the response rate, though they do not change the results [29].

### Target areas

The fact that only half of the responders indicated specific topics to be covered in patient surveys was particularly sobering since this might be another indicator for a certain disinterest in patient surveys, apart from low response rates. A potential explanation might be that patients do not expect a relevant impact from routine surveys or might be tired of ubiquitous consumer surveys [33].The responses to the open-ended question in the present survey clearly show that there is a variety of aspects that are important for patients in the context of healthcare provision. Despite variations in the terminology and definitions of the categories, our results agree with the NHS Patient Experience Framework. However, there are also some differences. Our respondents rarely mentioned friends and relatives in their comments, while the NHS Framework includes this aspect as a specific domain, ‘Welcoming the involvement of family and friends’. One explanation for this effect might be that our sample is likely dominated by outpatient contacts, for which the social environment might be of minor consequence. The aspects of friends and family might also differ between specialties and the duration of hospitalization. Another possible explanation is that our respondents subsumed this domain under more general comments on care, interactions with the staff or the atmosphere in the hospital.

In our sample the most frequently mentioned category is treatment. This contrasts with the NHS Framework, in which ‘treatment’ is not explicitly mentioned. This discrepancy might result from the assumption that patients might not be able to judge the medical quality of treatment adequately, but that patient-relevant aspects of treatment quality can rather be assessed by surrogate categories, e.g.,’ information, communication, and education’.

It is important to decide whether patients should be involved in the assessment of their treatment and, if so, in which way. In the category ‘Treatment’, the predominantly stated subcategories are ‘Treatment process’ and `Treatment outcomes and prognosis’. In this context ‘treatment process’ rather mirrors the efficacy of interdisciplinary functioning of healthcare provision, while ‘treatment outcomes and prognosis’ is a measure of patient management (quality of indication, communication and follow-up). Therefore, questions about these topics might be included in a patient survey. In this context, it should be said, that the high frequency of the category ‘treatment outcome’ might have been triggered by a preceding question, which explicitly mentions treatment outcomes as an aspect of patient surveys.

To benefit from a patient survey, the level of abstraction regarding questions on staff must be considered. The importance of the staff expressed by our respondents reflects the significance of human aspects in patient experience, perception and satisfaction as outlined in the literature [34]. Overall, our patients want to give feedback on staff in general or on the interaction with staff (communication and relationship building). They are less interested in commenting on the staff’s professional expertise perhaps because they feel less qualified to judge this aspect or they do not doubt it.

In the context of staff related survey preferences we interestingly found similar frequencies for the subcategories on ‘staff in general’ (i.e., general, communication and relationship building). In contrast, when considering the same subcategories for `physicians’ and ‘nursing staff’, we found distinct differences. `Physician–relationship building’ appeared less frequent than the two other subcategories of the physician category, while `nursing staff in general’ dominated over the other sub-categories in the nursing staff category. This lack of differentiation with respect to `Nursing staff’ might be caused by the connotations of the German word for nursing care (“Pflege”), which encompasses presumably female attributes such as empathy [35] as one aspect of relationship building. This illustrates the importance of clearly understanding and phrasing the aspects of interest in survey design e.g., the specific aspect of nursing care that patients should be referring to. In relation to physicians, most comments were about their communication, possibly pointing towards an area for improvement.

Finally it is important to note that patients do not seem to regard patient surveys as a place to openly express a desire for change. This underlines the need to analyze patient surveys carefully in order to identify any need for changes in patient management. Alternative approaches for involving patients in planning and evaluating procedural decisions might include targeting surveys on specific changes to be implemented, performing surveys before and after changes in management, or involving patient interest groups as stakeholders. Offering general patient surveys only on demand or only in small random subsamples (e.g. one day each month) may also lead to more informative feedback at reasonable cost.

### Limitations

The basic limitation of our study is that it was a survey with only one open-ended question. The responses to this question were often very unspecific, which suggests that this form of investigation may be suboptimal to elicit patient wishes. Future research should extend such investigations to include interviews or other techniques allowing to get more detailed information. Unfortunately, the anonymous nature of the survey precludes a structured nonresponder analysis. The low response rate and the low willingness to mention specific topics may be seen as limitations, or might reflect a putative lack of interest.

## Conclusion

In summary, our investigation challenges the current practice of routine patient surveys. The majority of patients seem to be rather indifferent with respect to the existence or content of patient surveys. They prefer broad topics to specific ones, and do not seem to regard patient surveys as a tool to make suggestions for improvement or initiate change. Hence we cannot expect to obtain detailed information, even if surveys are constructed according to the wishes of patients. Patients need to be engaged in a patient-centered and value-based healthcare system; therefore new approaches yielding potentially more meaningful results and better cost-efficacy need to be explored.

## Supporting information

S1 Table The classification system used. (PDF)

S2 Table The joint distribution of age and gender as reported by the participants. Shown is the absolute number for each possible combination including nonresponse to one of the two items. Total numbers are also given. Percentages refer to the number of subjects in the given age and gender group invited to participate in the survey.
(PDF)

S1 Fig The original questionnaire. Both the original German version as well as an translation into English are shown.
(PDF)

S2 Fig Distribution of choice of topics by age–main categories. This figure shows the relative frequency of participants mentioning a given main category within all participants of a specific age.
(PDF)

S3 Fig Distribution of choice of topics by gender–main categories. This figure showsthe relative frequency of participants mentioning a given main category within all participants of a specific gender.
(PDF)

S4 Fig Distribution of choice of topics by age–sub categories. This figure shows the relative frequency of participants mentioning a given sub category within all participants of a specific age.
(PDF)

S5 Fig Distribution of choice of topics by gender–sub categories. This figure showsthe relative frequency of participants mentioning a given sub category within all participants of a specific gender.
(PDF)

S1 Dataset The dataset used in the main analysis. (CSV)

S1 Dataset description A description of S1 Dataset. (DOCX)
