# Supplementary material for: Routine patient surveys: Patients’ preferences and information gained by healthcare providers
Source: PLoS One. 2019 Aug 1;14(8):e0220495. doi: 10.1371/journal.pone.0220495 (PMC6675389; doi:10.1371/journal.pone.0220495)
Supplement: S1 Table — (PDF) [file pone.0220495.s001.pdf]

Main categories,	Description:	Sub-Categories	Description	Example
Overall impression	Overall stay including the satisfaction and well-being of patients	Overall impression in general	Overall stay including the satisfaction and well-being of patients in general	Satisfaction<br>Hospital stay<br>Quality
		Specific departments	Overall impression of specific departments	Emergency Department<br>Physiotherapy
Administra-tion/ Organi-sation	Aspects regarding the organisation and administration of the hospital	Organisation	Overall organisation of the hospital and regulation of processes	Processes, Organisation<br>Visiting time
		Service	Features of the service including giving of appointments and the reception	Patient admission procedure<br>Adherence of appointments
		Waiting times	Waiting time before consultation and surgery	Waiting times<br>Duration between arrival and subsequent treatment
Staff	Aspects concerning the care by staff	Staff in general	Overall impression of staff and their care of patients	Care in the hospital
		Staff - communication	Provision of information and exchange between staff and patient. This includes information provision, consultation, explanations and verbal skills. In contrast to the sub-category 'Staff - relationship building', this category refers primarily to the verbal aspects of communication.	Informing patient<br>Comprehensibility<br>Clarity / transparency
		Staff - relationship building	Aspects relating to the manners (e.g. contact, friendliness) and the patient-staff-relationship (trust, being responsive to needs). They can include verbal communication but can also be displayed in a different manner. Comments that are clearly referring to verbal communication are classified into the sub-category `Staff-communication'.	Friendliness<br>“(create) trust!?”
		Staff - professional expertise	Professional knowledge and skills of the staff	Professional expertise<br>Quality of professional help

		Staff - continuity	(Non-)continuity of staff	(Changing) contact<br>Do you like to get a different part-time employee every day?
Nursing staff	Aspects concerning the care by the nursing staff	Nursing staff in general	Overall impression of nursing staff and their care of patients	Quality of care by nursing staff<br>Nursing staff
		Nursing staff - communication	Provision of information and exchange between nursing staff and patient. This includes information provision, consultation, explanations and verbal skills. In contrast to the sub-category 'nursing staff - relationship building', this category refers primarily to the verbal aspects of communication.	Nursing staff (communication)
		Nursing staff - Relationship building	Aspects relating to the manners (e.g. contact, friendliness) and the patient-nurse-relationship (trust, being responsive to needs). They can include verbal communication but can also be displayed in a different manner. Comments that are clearly referring to verbal communication are classified into the sub-category 'nursing staff-communication'.	Nursing team (How was the atmosphere? Were the nurses empathetic?)
		Nursing staff - professional expertise	Professional knowledge and skills of the nursing staff	Competence of nursing staff<br>Professional expertise of nursing staff
Physicians and Surgeons	Aspects concerning the care by the physicians and surgeons	Physicians in general	Overall impression of physicians and surgeons and their care of patients	Medical care<br>Attending physician
		Physicians - communication	Provision of information and exchange between physician and patient. This includes information provision, consultation, explanations and verbal skills. In contrast to the sub-category 'Physician - relationship building', this category refers primarily to	Comprehensible information regarding the current problem as well as treatment options and the selected treatment including treatment outcome<br>Information provision by the physician

			the verbal aspects of communication.	
		Physicians - Relationship building	Aspects relating to the manners (e.g. contact, friendliness) and the patient-physician-relationship (trust, being responsive to needs). They can include verbal communication but can also be displayed in a different manner. Comments that are clearly referring to verbal communication are classified into the sub-category 'physician-communication'.	Patient-physician-relationship<br>Trust in physicians
		Physicians - professional expertise	Professional knowledge and skills of the physicians and surgeons	Physicians' qualifications<br>Medical expertise
Collabora-tion	Collaboration within the hospital and with entities outside the hospital			Collaboration between the medical departments<br>Handover to GP<br>Surgery Bethesda - university hospital - GP
Treatment	Aspects of the treatment	Medical characteristics	Health status and medical data	Reason for treatment<br>General condition<br>Diagnosis
		Treatment process	Aspects of medical and therapeutic interventions	Satisfaction with treatment<br>Quality of treatment
		Treatment outcome + prognosis	Treatment outcome and prognosis and satisfaction with them	Outcomes of treatment<br>Outcome quality of the treatment
		Follow-up care	Care following surgery and hospital stay	Post-treatment care following the operation
Additional hospital service	Hospital services which are not related to medical treatment and nursing	Gastronomy	Aspects of catering	Food<br>Meals
		Accommodation	Aspects of accommodation	Rooms<br>Comfort
		Infrastructure	General facilities of the hospital	Infrastructure<br>Parking

		Hygiene	Aspects of cleanliness	Cleanliness<br>Hospital hygiene
Costs	Financial aspects and efficiency			Costs of health insurance<br>Was the treatment worth its costs?
Suggestions for im-provement	Opportunity to give comments and suggestions for improvement			requests for change<br>Suggestions for improvement
Incomprehe nsible	Comment was incomprehensible			
