# Supplementary material for: Routine patient surveys: Patients’ preferences and information gained by healthcare providers
Source: PLoS One. 2019 Aug 1;14(8):e0220495. doi: 10.1371/journal.pone.0220495 (PMC6675389; doi:10.1371/journal.pone.0220495)
Supplement: S2 Table — Shown is the absolute number for each possible combination including nonresponse to one of the two items. Total numbers are also given. Percentages refer to the number of subjects in the given age and gender group invited to participate in the survey. (PDF) [file pone.0220495.s002.pdf]

	gender			
age	female (%of total contacted)	male (%of total contacted)	unknown	total
<20	4 (10.3%)	3 (5.7%)	2	9
20 - 30	10 (10.2%)	17 (9.6%)	2	29
31 - 40	17 (12.5%)	20 (8.4%)	7	44
41 - 50	48 (23.4%)	38 (15.7%)	13	99
51 - 60	55 (21.7%)	50 (18.0%)	11	116
61 - 70	82 (35.5%)	61 (29.5%)	11	154
71 - 80	89 (33.0%)	46 (31.7%)	15	150
81 - 90	50 (26.0%)	39 (42.9%)	10	99
> 90	20 (31.7%)	4 (28.6%)	2	26
unknown	1	0	18	19
total	376(25.3%)	278 (19.3%)	91	745
