# Supplementary material for: Routine patient surveys: Patients’ preferences and information gained by healthcare providers
Source: PLoS One. 2019 Aug 1;14(8):e0220495. doi: 10.1371/journal.pone.0220495 (PMC6675389; doi:10.1371/journal.pone.0220495)
Supplement: S1 Fig — Both the original German version as well as an translation into English are shown. (PDF) [file pone.0220495.s003.pdf]

Bitte füllen Sie diesen Fragebogen aus und senden Sie diesen mit dem beigelegten Rückantwortcouvert bis zum 15.07.2017 kostenfrei an uns zurück.

Herzlichen Dank für Ihre Unterstützung!

Demographische Angaben:

Alter:	jünger als 20 Jahre ☐<br>20-30 Jahre ☐<br>31-40 Jahre ☐<br>41-50 Jahre ☐<br>51-60 Jahre ☐	61-70 Jahre ☐<br>71-80 Jahre ☐<br>81-90 Jahre ☐<br>älter als 90 Jahre ☐<br>keine Angabe ☐

Geschlecht:	Weiblich ☐<br>Männlich ☐<br>Keine Angabe ☐

---

	ja	nein	keine Angabe
**1. Möchten Sie vom Universitätsspital zu Ihrem Spitalaufenthalt befragt werden?**	☐	☐	☐

Wenn ja, zu welchen Inhalten möchten Sie befragt werden?

a. zum Ergebnis Ihrer Behandlung? ☐

b. zu Ihrer Zufriedenheit mit dem Universitätsspital? ☐

c. zu beiden Aspekten (Behandlungsergebnis und Zufriedenheit)? ☐

2. In welcher Form würden Sie sich eine Befragung wünschen?
(Mehrfachnennungen möglich)

- ☐ per SMS
- ☐ per App
- ☐ per Email
- ☐ online im Internet
- ☐ in Briefform

3. Bitte nennen Sie uns 3 Themengebiete, die Sie in einer Patientenbefragung wichtig finden:

_1.__________________________________

_2.__________________________________

_3.__________________________________

4. Und noch eine letzte Frage:
Wie viele Befragungsbögen haben Sie in den letzten 6 Monaten vom Universitätsspital bekommen (diesen hier eingerechnet): ______ Anzahl

Please fill out this questionnaire and return it to us free of charge until July 15, 2017 using the enclosed reply envelope.

Thank you very much for your cooperation!

Demographics:

Age:	under 20 years ☐<br>20-30 years ☐<br>31-40 years ☐<br>41-50 years ☐<br>51-60 years ☐	61-70 years ☐<br>71-80 years ☐<br>81-90 years ☐<br>older than 90 years ☐<br>no information ☐

Gender:	Female ☐<br>Male ☐<br>No information ☐

	Yes	No	No information
**1.** Would you like to be surveyed about your hospital stay by the University Hospital?	☐	☐	☐

If so, what content would you like to be asked about?

a. about the result of your treatment? ☐

b. about your satisfaction with the University Hospital? ☐

c. about both aspects (Treatment result and satisfaction)? ☐

2. In what form would you like to be surveyed?
(Multiple answers are allowed)

- ☐ via SMS
- ☐ via app
- ☐ via email
- ☐ online (internet)
- ☐ by letter

3. Please name 3 topics that you find important in a patient survey:

_1.________________________________

_2.________________________________

_3.________________________________

4. And a final question:
How many questionnaires have you received from the University Hospital in the last 6 months (including this one): ______ Number
