# Supplementary figures and images for: Routine patient surveys: Patients’ preferences and information gained by healthcare providers
Source: PLoS One. 2019 Aug 1;14(8):e0220495. doi: 10.1371/journal.pone.0220495 (PMC6675389; doi:10.1371/journal.pone.0220495)

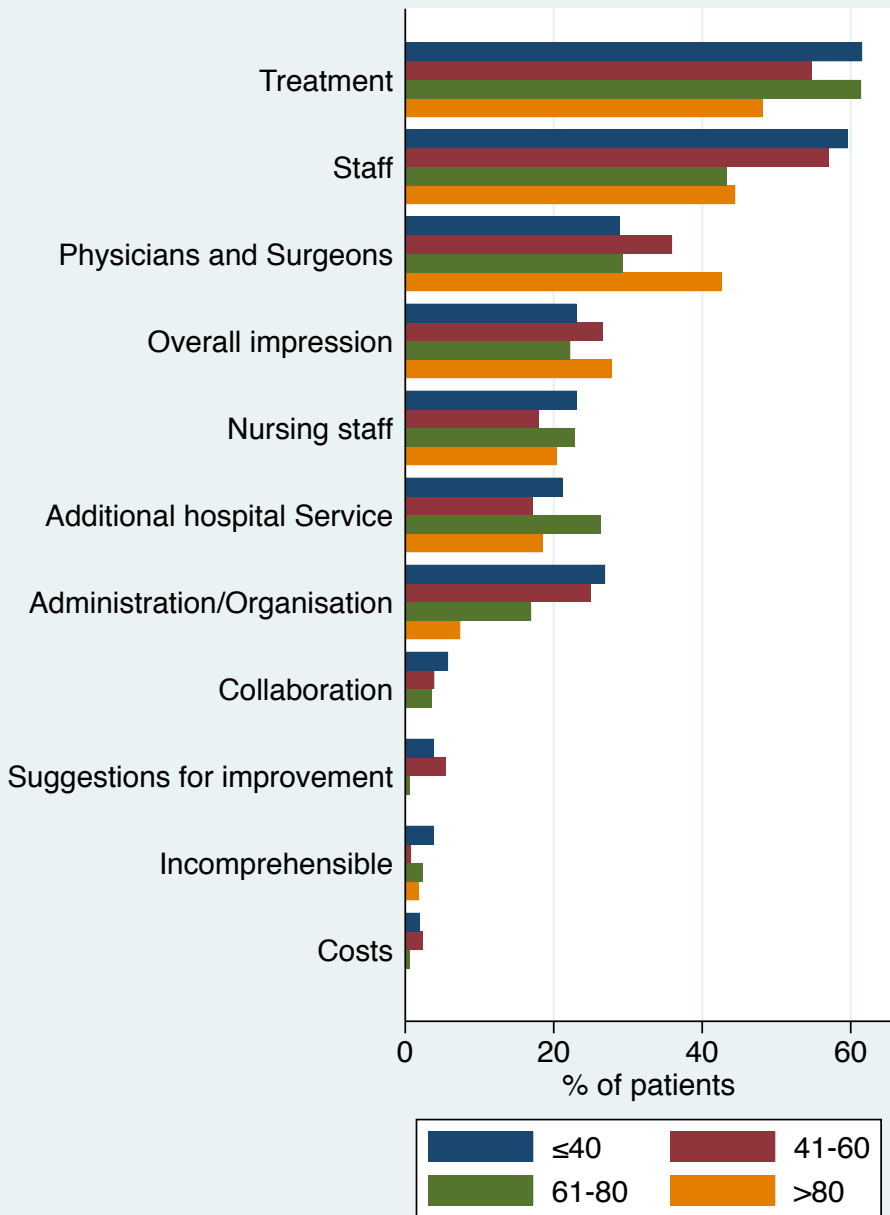

Supplement: S2 Fig — This figure shows the relative frequency of participants mentioning a given main category within all participants of a specific age. (PDF) [file pone.0220495.s004.pdf]

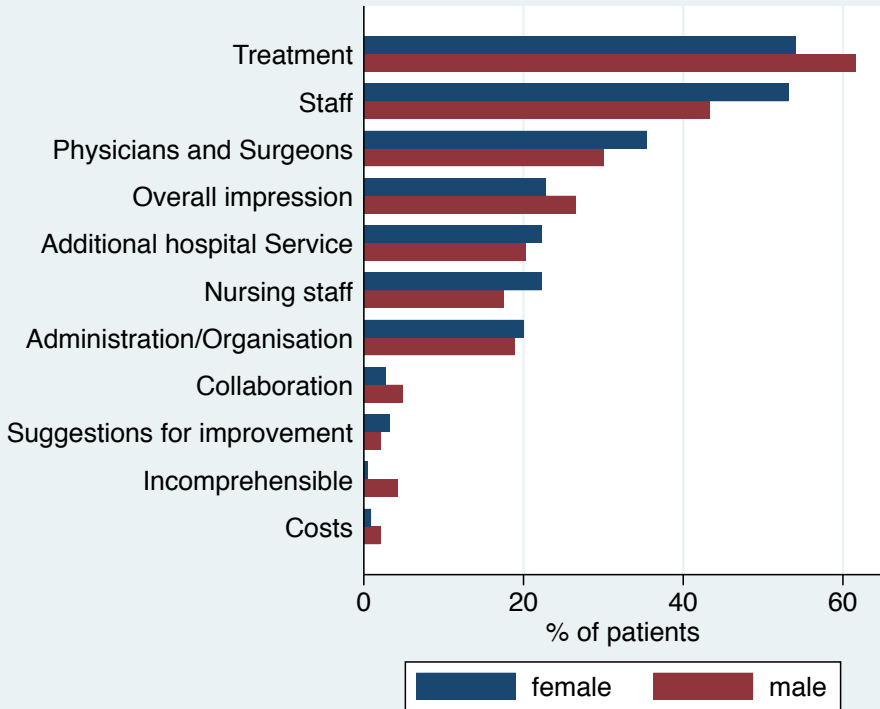

Supplement: S3 Fig — This figure showsthe relative frequency of participants mentioning a given main category within all participants of a specific gender. (PDF) [file pone.0220495.s005.pdf]

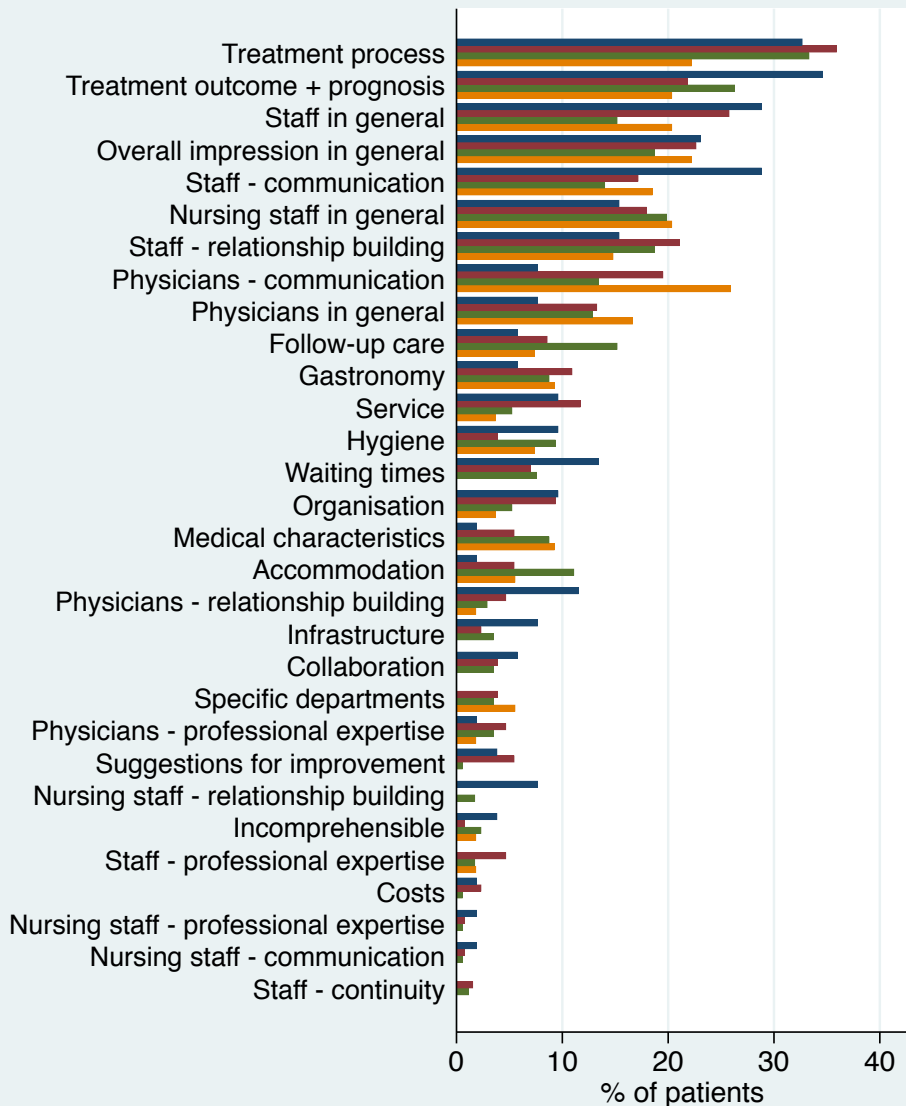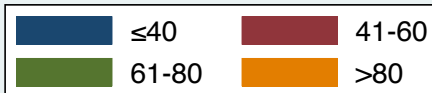

Supplement: S4 Fig — This figure shows the relative frequency of participants mentioning a given sub category within all participants of a specific age. (PDF) [file pone.0220495.s006.pdf]

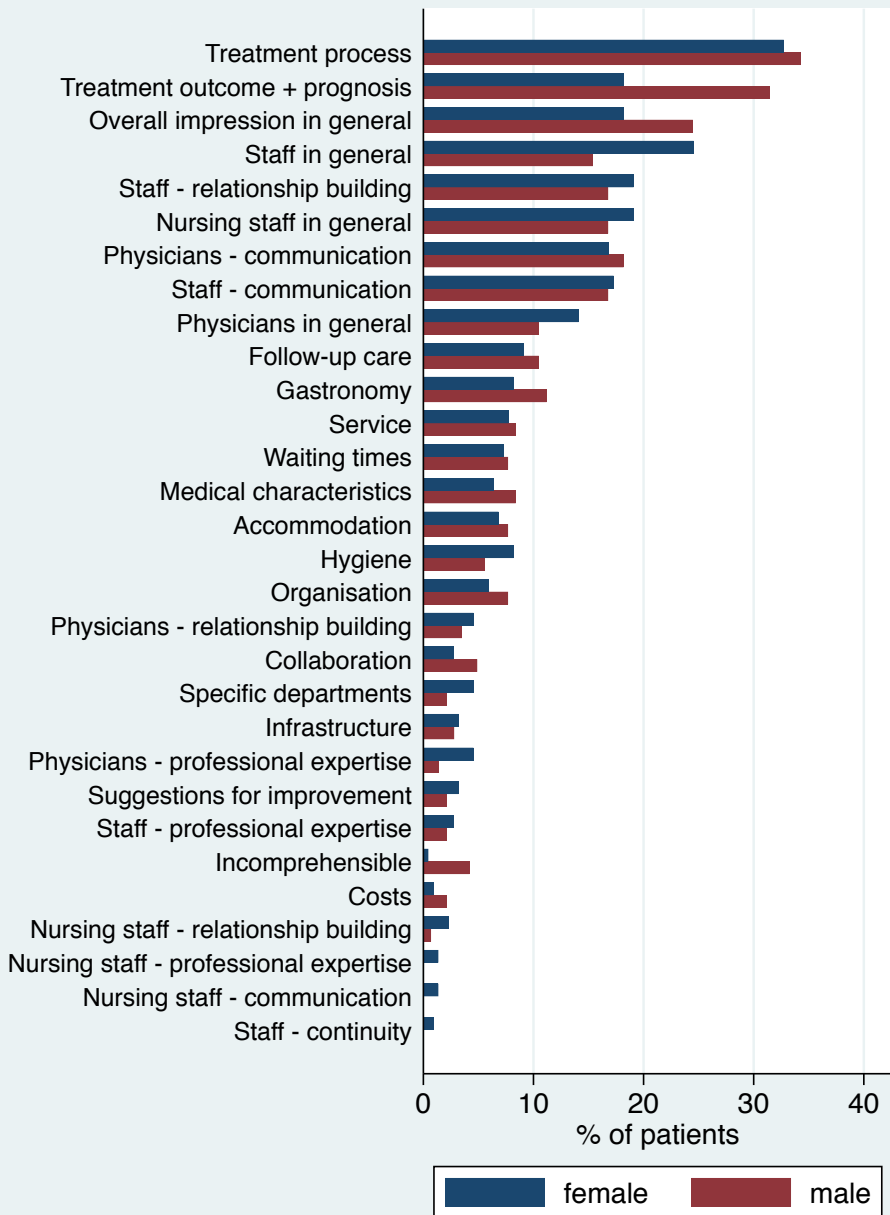

Supplement: S5 Fig — This figure showsthe relative frequency of participants mentioning a given sub category within all participants of a specific gender. (PDF) [file pone.0220495.s007.pdf]
