# Supplementary material for: Routine patient surveys: Patients’ preferences and information gained by healthcare providers
Source: PLoS One. 2019 Aug 1;14(8):e0220495. doi: 10.1371/journal.pone.0220495 (PMC6675389; doi:10.1371/journal.pone.0220495)
Supplement: S1 Dataset description — (DOCX) [file pone.0220495.s009.docx]

| Variable name | Explanation | Coding |
| --- | --- | --- |
| record_id | Patient number (arbitrarily chosen) | number |
| answer | First, second or third answer | 1 ,2,3 |
| age_groups | Age_groups | 1: <=40; 2: 41-60; 3: 61-80; 4: 80 |
| gender | Gender | 1=female; 2=male |
| like_to_be_asked | Question: Would you like to be surveyed about your hospital stay  by the University Hospital? | 0/1 |
| preferred_focus | Question: If so, what content would you like to be asked about? | 1=treatment 2=satisfaction 3=both |
|  | *Questions on contact form* |  |
| contact_sms | via SMS (text message) | 0/1 |
| contact_app | via app | 0/1 |
| contact_email | via email | 0/1 |
| contact_online | Online (internet) | 0/1 |
| contact_letter | by letter | 0/1 |
| number_of_surveys | Number of surveys obtained in the last 6 months | number |
|  | *Sub categories* |  |
| SubOverallimpressioningeneral | Overall impression in general | 0/1 |
| SubSpecificdepartments | Specific departments | 0/1 |
| SubOrganisation | Organisation | 0/1 |
| SubService | Service | 0/1 |
| SubWaitingtimes | Waiting times | 0/1 |
| SubStaffingeneral | Staff in general | 0/1 |
| SubStaffcommunication | Staff - communication | 0/1 |
| SubStaffrelationshipbuilding | Staff - relationship building | 0/1 |
| SubStaffprofessionalexpertise | Staff - professional expertise | 0/1 |
| SubStaffcontinuity | Staff - continuity | 0/1 |
| SubNursingstaffingeneral | Nursing staff in general | 0/1 |
| SubNursingstaffcommunication | Nursing staff - communication | 0/1 |
| SubNursingstaffrelationshipbui | Nursing staff – relationship building | 0/1 |
| SubNursingstaffprofessionalexp | Nursing staff – professional expertise | 0/1 |
| SubPhysiciansingeneral | Physicians in general | 0/1 |
| SubPhysicianscommunication | Physicians - communication | 0/1 |
| SubPhysiciansrelationshipbuildi | Physicians - relationship building | 0/1 |
| SubPhysiciansprofessionalexpert | Physicians – professional expertise | 0/1 |
| SubCollaboration | Collaboration | 0/1 |
| SubMedicalcharacteristics | Medical characteristics | 0/1 |
| SubTreatmentprocess | Treatment process | 0/1 |
| SubTreatmentoutcomeprognosis | Treatment outcome + prognosis | 0/1 |
| SubFollowupcare | Follow-up care | 0/1 |
| SubGastronomy | Gastronomy | 0/1 |
| SubAccommodation | Accommodation | 0/1 |
| SubInfrastructure | Infrastructure | 0/1 |
| SubHygiene | Hygiene | 0/1 |
| SubCosts | Costs | 0/1 |
| SubSuggestionsforimprovement | Suggestions for improvement | 0/1 |
| SubIncomprehensible | Incomprehensible | 0/1 |
|  | *Main categories* |  |
| CatOverallimpression | Overall impression | 0/1 |
| CatAdministrationOrganisation | Administration/Organisation | 0/1 |
| CatStaff | Staff | 0/1 |
| CatNursingstaff | Nursing staff | 0/1 |
| CatPhysiciansandSurgeons | Physicians and Surgeons | 0/1 |
| CatCollaboration | Collaboration | 0/1 |
| CatTreatment | Treatment | 0/1 |
| CatAdditionalhospitalService | Additional Hospital Service | 0/1 |
| CatCosts | Costs | 0/1 |
| CatSuggestionsforimprovement | Suggestions for improvement | 0/1 |
| CatIncomprehensible | Incomprehensible | 0/1 |
